# The Antiangiogenic 16K Prolactin Impairs Functional Tumor Neovascularization by Inhibiting Vessel Maturation

**DOI:** 10.1371/journal.pone.0027318

**Published:** 2011-11-07

**Authors:** Ngoc-Quynh-Nhu Nguyen, Karolien Castermans, Sarah Berndt, Stephanie Herkenne, Sebastien P. Tabruyn, Silvia Blacher, Michelle Lion, Agnes Noel, Joseph A. Martial, Ingrid Struman

**Affiliations:** 1 Unit of Molecular Biology and Genetic Engineering, GIGA-Research, University of Liège, Liège, Belgium; 2 Laboratory of Biology of Tumor and Development, GIGA-Research, University of Liège, Liège, Belgium; Children's Hospital & Harvard Medical School, United States of America

## Abstract

**Background:**

Angiogenesis, the formation of new blood vessels from existing vasculature, plays an essential role in tumor growth, invasion, and metastasis. 16K hPRL, the antiangiogenic 16-kDa N-terminal fragment of human prolactin was shown to prevent tumor growth and metastasis by modifying tumor vessel morphology.

**Methodology/Principal Findings:**

Here we investigated the effect of 16K hPRL on tumor vessel maturation and on the related signaling pathways. We show that 16K hPRL treatment leads, in a murine B16-F10 tumor model, to a dysfunctional tumor vasculature with reduced pericyte coverage, and disruption of the PDGF-B/PDGFR-B, Ang/Tie2, and Delta/Notch pathways. In an aortic ring assay, 16K hPRL impairs endothelial cell and pericyte outgrowth from the vascular ring. In addition, 16K hPRL prevents pericyte migration to endothelial cells. This event was independent of a direct inhibitory effect of 16K hPRL on pericyte viability, proliferation, or migration. In endothelial cell-pericyte cocultures, we found 16K hPRL to disturb Notch signaling.

**Conclusions/Significance:**

Taken together, our data show that 16K hPRL impairs functional tumor neovascularization by inhibiting vessel maturation and for the first time that an endogenous antiangiogenic agent disturbs Notch signaling. These findings provide new insights into the mechanisms of 16K hPRL action and highlight its potential for use in anticancer therapy.

## Introduction

Angiogenesis is a crucial step in many pathologies including tumor growth and metastasis [1]. Antiangiogenic therapy is thus considered as an important option for the treatment of cancer. The 16-kDa N-terminal fragment of human prolactin (16K hPRL) has been shown to impair angiogenesis both *in vitro* and *in vivo*. In particular, 16K hPRL inhibits the proliferation of endothelial cells [2], [3] (both basal and stimulated by basic fibroblast growth factor (bFGF) and vascular endothelial growth factor (VEGF)) by blocking endothelial cell cycle in the G_0_–G_1_ and G_2_-M transitions through combined effects on positive and negative regulators of cell cycle progression: it down regulates cyclin-D1 and cyclin-B1 and up regulates p21cip1 and p27kip1 [4]. It has been also demonstrated that 16K hPRL induces endothelial cell apoptosis by activating Caspase-3 and NF-κB [5], [6]. NF-κB is necessary for activation of Caspase-8 and -9, which in turn trigger Caspase-3 activation and DNA fragmentation [6]. In addition, 16K hPRL inhibits endothelial cell migration by downregulating the Ras-Tiam1-Rac1-Pak1 signaling pathway [7]. The antiangiogenic activity of 16K hPRL appears to be mediated by a saturable high-affinity binding site distinct from the PRL receptor [8]. *In vivo*, excess of 16K hPRL has been shown to mediate human postpartum cardiomyopathy (PPCM) [9]. Treatment with bromocriptine, an inhibitior of prolactin secretion, prevents the development of PPCM in mice, whereas forced myocardial generation of 16K hPRL impairs the cardiac capillary network and function, thereby recapitulating the cardiac phenotype of PPCM. Finally, 16K hPRL has been shown to inhibit angiogenesis in a mouse retinopathy model [10], and to prevent tumor growth and metastasis establishment in various mouse models [11], [12], [13], [14]. Tumors of 16K hPRL-treated mice are characterized by an increased number of vessels that are, however, smaller and narrower [13], [14]. In addition, in tumor tissues a higher expression of adhesion molecules (ICAM-1, VCAM-1, or E-selectin) in endothelial cells and a significantly higher number of infiltrated leucocytes is observed within the tumor treated with 16K hPRL compared with the untreated ones [15].

The maturation of new vasculature requires pericyte recruitment, generation of an extracellular matrix, and specialization of the vessel wall for structural support and regulation of vessel function. These processes are orchestrated by a constellation of ligands and receptors on pericytes and endothelial cells, whose spatio-temporal patterns of expression and concentration are tightly regulated. Inappropriate levels of these molecules produce an abnormal vasculature, a hallmark of various pathologies [16].

Several signaling pathways, such as the PDGF-B/PDGFR-B, Ang/Tie2, TGF-β, sphingosine-1-phosphate (S1P), Notch, and EphrinB2 pathways, have been associated with pericyte recruitment and heterotypic communication between endothelial cells and pericytes [17]. PDGF-B is secreted by endothelial cells to attract comigrating pericytes expressing its receptor, PDGFR-B. It also stimulates proliferation of vascular smooth muscle cells and induces a mural cell fate in undifferentiated mesenchymal cells [16], [18], [19]. Ang1 expressed by mural cells binds to and activates the Tie2 receptor on endothelial cells. Consequently, angiogenesis is stimulated by increased endothelial cell survival and subsequent mural cell attachment. The role of Ang1 in tumor-associated angiogenesis remains controversial and depends on the tumor cell type and/or the dosage of Ang2 in the tumors [20]. The role of Ang2 appears to be contextual. In the absence of VEGF, endothelium-derived Ang2 is an antagonistic ligand of Tie2, causing endothelial cell death and defective maturation. Ang2 expression appears to be negatively correlated with pericyte coverage in both spontaneous and experimental tumor models [16], [18], [19], [21]. In contrast to the PDGF-B/PDGFR-B and Ang/Tie2 signaling pathways allowing cell-contact-independent endothelial/mural cell crosstalk, the Notch and EphrinB2 signaling pathways require direct endothelial cell-mural cell contact. Notch receptors (Notch1 to 4), their ligands (Delta-like and Jagged), and their downstream mediators (Hes/Hey) are highly expressed, in different combinations, in both endothelial cells and mural cells. Notch3, the predominant Notch receptor in vascular smooth muscle cells, can be activated in endothelial cells by Dll1 and Jagged1, thereby promoting mural cell maturation [22], [23], [24]. Evidence of increased Dll4 expression in the tumor endothelium correlates with tumor vessel maturation and remodeling [25], [26]. EphrinB2, a ligand of Eph receptor tyrosine kinases, has recently been characterized as a key mediator in the assembly of vascular structures by endothelial and mural cells [27], [28]. By interacting with Notch4, Dll4 can upregulate the expression of EphrinB2 in endothelial cells under both physiological and pathological conditions [25], [29].

As stated above, tumor growth suppression by 16K hPRL is accompanied by an increased tumor vessel density and the appearance of small and narrow, collapsed capillaries. This specific phenotype led us to hypothesize that 16K hPRL might prevent the development of a functional tumor vasculature by affecting vessel maturation. Here, we provide evidence in favor of this hypothesis.**** We demonstrate, firstly, that in the murine B16-F10 melanoma model, 16K hPRL treatment decreases tumor perfusion and reduces pericyte coverage of tumor vessels. We further describe *ex vivo* and *in vitro* experiments showing that 16K hPRL does not affect pericytes directly but causes defective pericyte recruitment to endothelial cells with disruption of the PDGF-B, Ang, and Notch pathways.

## Results

### 16K hPRL reduces vessel perfusion and pericyte coverage of tumor vasculature

**Figure 1:**
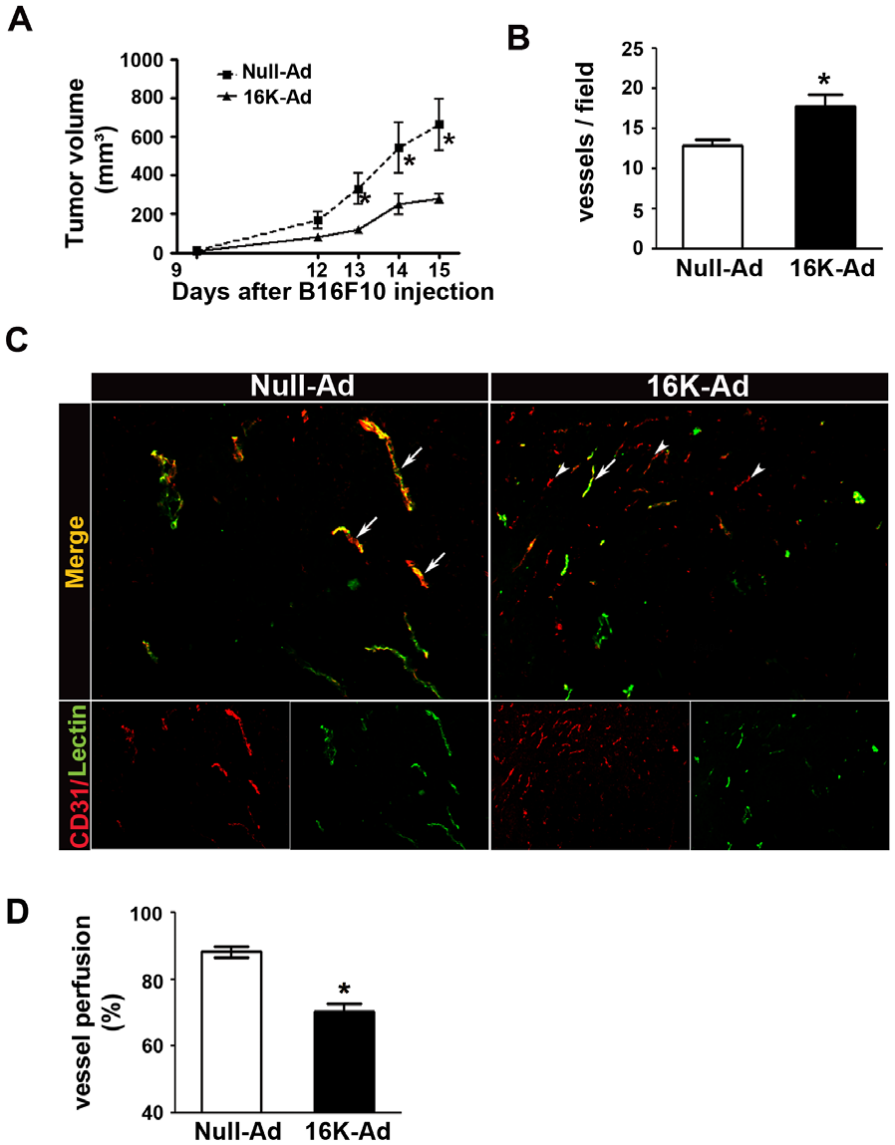
Inhibition of tumor growth by 16K hPRL is associated with an increased vessel number but with decreased vessel perfusion. (A) The data represent mean tumor volumes ± SEM in 16K-Ad-treated (n = 8) or Null-Ad-treated (n = 10) mice. (B) The number of tumor vessels per field ± SEM was determined on CD31-positive vessels in x100 power fields of each section. *Bar*, 100 µm. (C) Representative photographs of tumor sections from 16K-Ad- and Null-Ad-inoculated mice, double-stained for the endothelial cell marker CD31 (red) and the perfusion marker lectin (green). White arrows, vessels stained positive for CD31 and lectin. Heads of white arrows, vessels only positive for CD31. (D) The perfusion index was calculated as the percentage of lectin-positive vessels (perfused vessels) per CD31-positive vessels in the B16F10 tumors ± SEM. For (B) and (D) the data represent, for each set of conditions, the mean ratio ± SEM calculated for all the fields of all tumor sections (one section per tumor). *, P<0.05.

**Figure 2:**
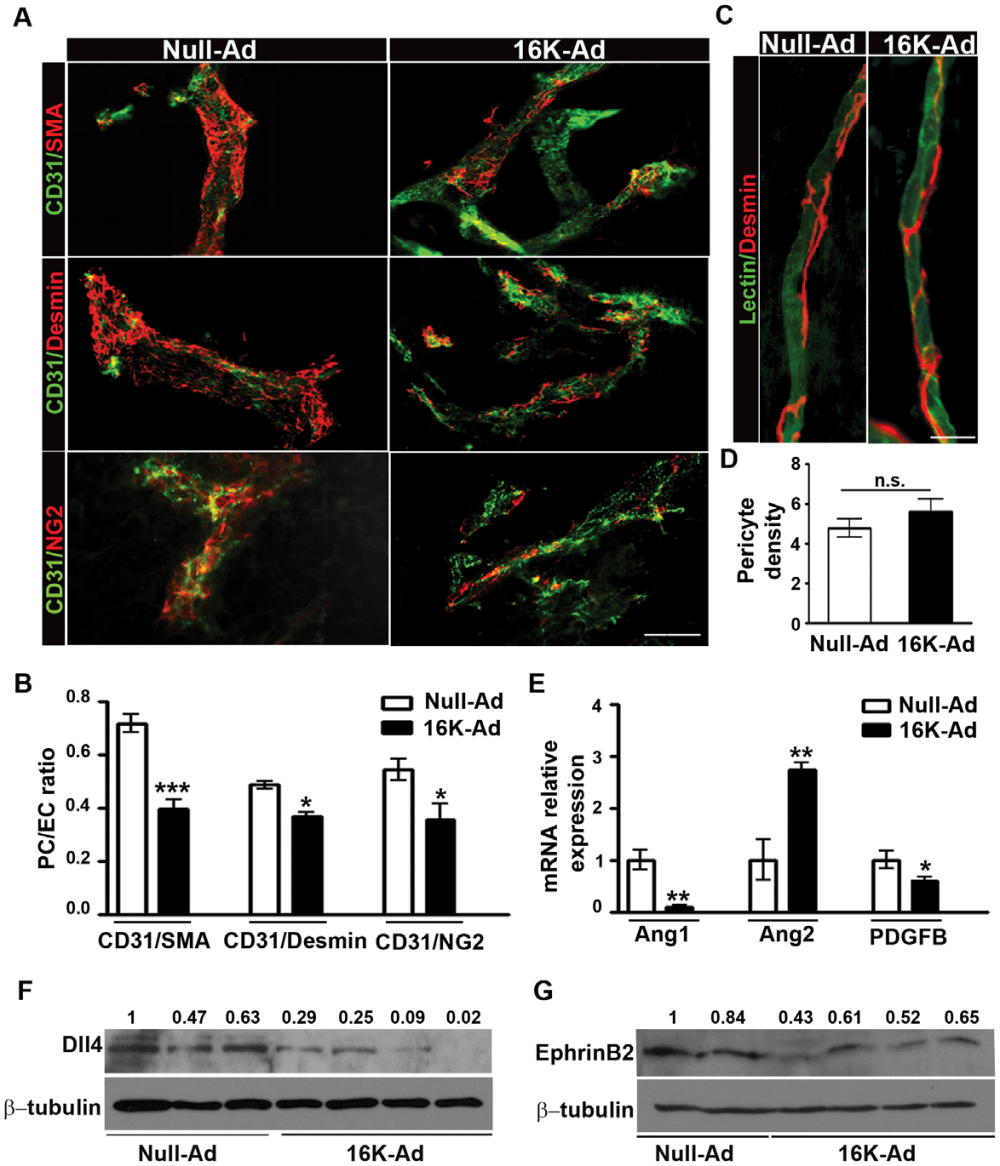
Reduced pericyte coverage in B16F10 tumor vessels after 16K-Ad treatment. (A) Representative photographs of 80 µm thick tumor sections from 16K-Ad- and Null-Ad-inoculated mice, double-stained for the endothelial cell marker CD31 (green) and the mural cell markers α-SMA, desmin, and NG2 (red). (B) Computer-assisted image analysis was used to quantify pericyte coverage. The area occupied by vessels and the covered area were estimated. Coverage was calculated by dividing the latter area by the former. The data represent, for each set of conditions, the mean ratio ± SEM calculated for all the fields of all tumor sections (one section per tumor). We analyzed 10 tumors of Null-Ad-treated and 8 tumors of 16K-Ad-treated mice. *Bar*, 100 µm. (C) Representative photographs of tracheal capillaries. Tracheal capillaries from 16K-Ad- and Null-Ad-inoculated mice were visualized by lectin-FITC staining (green) and pericytes were visualized by desmin immunostaining (red). *Bar*, 25 µm. (D) Computer-assisted image analysis was used to quantify pericyte density. (E) RNAs were extracted from tumors and the relative abundance ± SEM of each specified mRNA (encoding pdgf-b, Ang1, or Ang2) was assessed by qRT-PCR in 10 Null-Ad tumors and 8 16K-Ad tumors. (F) Western blot analysis of Dll4 and (G) EphrinB2 in different 16K-Ad-treated and control tumors. n.s., non significant. *, P<0.05. **, P<0.001. ***, P<0.0001.

In the murine subcutaneous B16F10 melanoma model, the antiangiogenic 16K hPRL significantly reduced tumor growth by about 50%, but increased the vessel density as previously shown [13] (Figure 1A–B). These observations led us to hypothesize that 16K hPRL impairs the functionality of the tumor vascular network rather than reducing vessel density. To test this hypothesis, we examined the effect of 16K hPRL on tumor vessel perfusion by comparing the distribution of intravascular lectin (used as a tracer of perfused blood vessels) with that of CD31 (used to mark all tumor vessels). We observed about 25% less perfusion in the tumors of 16K-Ad-treated mice than in those of control mice (Figure 1C–D). We wondered what might contribute to impairing vascular function in the tumors of 16K-Ad-treated mice. Given the small, narrow capillaries that characterize these tumors [13], [14], we hypothesized that 16K hPRL might prevent vessel maturation. To test this hypothesis and because vessel maturation requires the recruitment of pericytes, we first examined the mural cell coverage of tumor vessels by immunohistochemical staining. Whatever mural marker used (αSMA, desmin, or NG2), we observed a significantly (20 to 40%) decreased coverage of CD31-positive blood vessels by mural cells after 16K hPRL treatment (Figure 2A–B). This effect of 16K hPRL was tumor-vessel specific, since we detected no decrease in mural cell coverage and no reduction of vessel perfusion in tracheal capillaries from the 16K-Ad-treated mice (Figure 2C-D).

Next, we used qRT-PCR to measure the levels of transcripts of two genes (*Pdgfb* and *Ang1*) encoding positive regulators and one gene (*Ang2*) encoding a negative regulator of vessel maturation. In tumors of Ad-16K treated mice, the levels of *Pdgfb* and *Ang1* mRNAs were respectively 1.7-fold and 10-fold lower than in tumors of control mice, whereas the level of *Ang2* transcripts was 3-fold higher (Figure 2E). We then used Western blotting to estimate levels of Dll4, a Notch ligand known to be upregulated during tumor angiogenesis [30], [31], and EphrinB2, a downstream target of Dll4. The levels of both proteins are reduced in the tumors of Ad-16K treated mice (Figure 2F–G).

Together, these results support the view that 16K hPRL impairs tumor vessel maturation in our mouse model.

### 16K hPRL inhibits pericyte outgrowth in an *ex vivo* murine aortic ring assay

**Figure 3:**
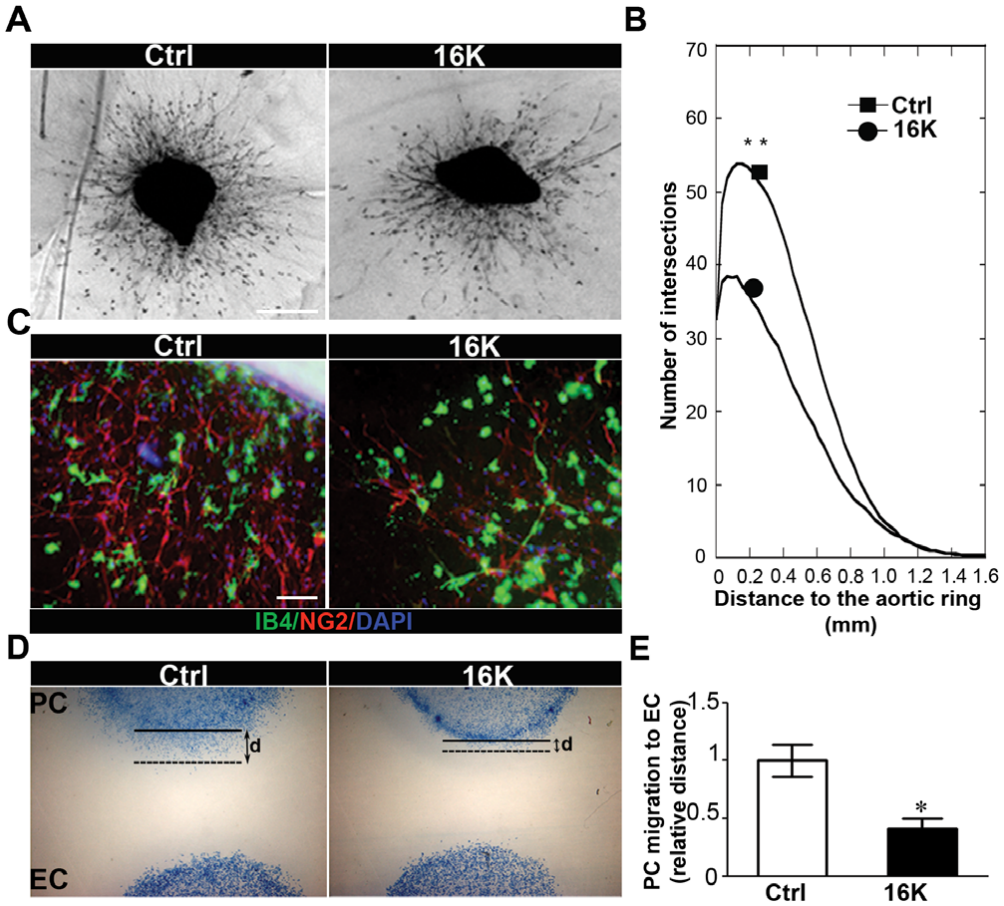
16K hPRL inhibits pericyte outgrowth in an aortic ring assay and pericyte migration towards endothelial cells. (A) Photomicrographs of mouse aortic ring cultured in collagen for 9 days and incubated without specific treatment (Ctrl) or under 16K hPRL treatment (16K). Data representative of at least 2 independent experiments are shown. *Bar*, 1 mm. (B) Quantification of migrating cell distribution around the aortic ring performed by computerized image analysis. Cell distribution is defined as the number of intersections between spreading cells and a grid of concentric rings, plotted as a function of the distance to the aortic fragment. Each curve is a mean of the cell network distribution obtained by averaging at least 5 individual distributions generated for each experimental condition. (C) Photomicrographs of a mouse aortic ring cultured in collagen for 9 days without specific treatment (Ctrl) or in the presence of 100 nM 16K hPRL. The rings were fixed for subsequent immunostaining: anti-Isolectin B4 Ab (IB4: green) identifying EC, anti-NG2 proteoglycan Ab (NG2: red) identifying PC/SMC. Nuclei were colored with DAPI (blue). Data representative of at least 2 independent experiments are shown. *Bar*, 100 µm. (D) HBVP migration towards HUVEC was assessed in an under-agarose migration coculture assay. HUVEC were treated for 72 h with 100 nM 16K hPRL (16K). d = migration distance between the beginning (solid line) and the end of the migration (dotted line). *, P<0.05.

To further scrutinize the effects of 16K hPRL on vascular maturation and morphogenesis, we used the murine aortic ring assay. This assay measures the formation, from the aortic ring, of a new network consisting of IB4-positive endothelial cells and NG2-positive pericytes/smooth muscle cells (PC/SMC). Mouse aortic ring explants were incubated with or without recombinant 16K hPRL, and the angiogenic response was observed under bright field microscopic examination (Figure 3A) and quantified by computer-assisted image analysis for the length of the tube (Figure 3B). In the presence of 16K hPRL, cellular network formation was inhibited and fewer NG2-positive cells were observed (Figure 3C). Thus, the antiangiogenic protein 16K hPRL inhibits the outgrowth of a PC/SMC network.

### 16K hPRL inhibits pericyte recruitment by endothelial cells *in vitro*

Next, we examined whether the observed inhibition of pericyte network development by 16K hPRL might be due to defective pericyte recruitment to endothelial cells. In an under-agarose migration assay, we measured the ability of HBVP to migrate towards HUVEC in the presence and absence of 16K hPRL (Figure 3D–E). Each cell type was placed in one of two wells made 1 mm apart, the HBVP were allowed to migrate for 72 h, and their migration distance was measured. HBVP cultured in the absence of HUVEC exhibited a pattern of random movement, migrating uniformly from all edges of the circle. When HBVP were cocultured with HUVEC for 72 h, they migrated towards the HUVEC in a directional manner. In the presence of 16K hPRL, the HBVP migration distance was about 50% shorter than in its absence. These results indicate that 16K hPRL prevents pericyte recruitment to endothelial cells.

### 16K hPRL acts specifically on endothelial cells but not on pericytes

**Figure 4:**
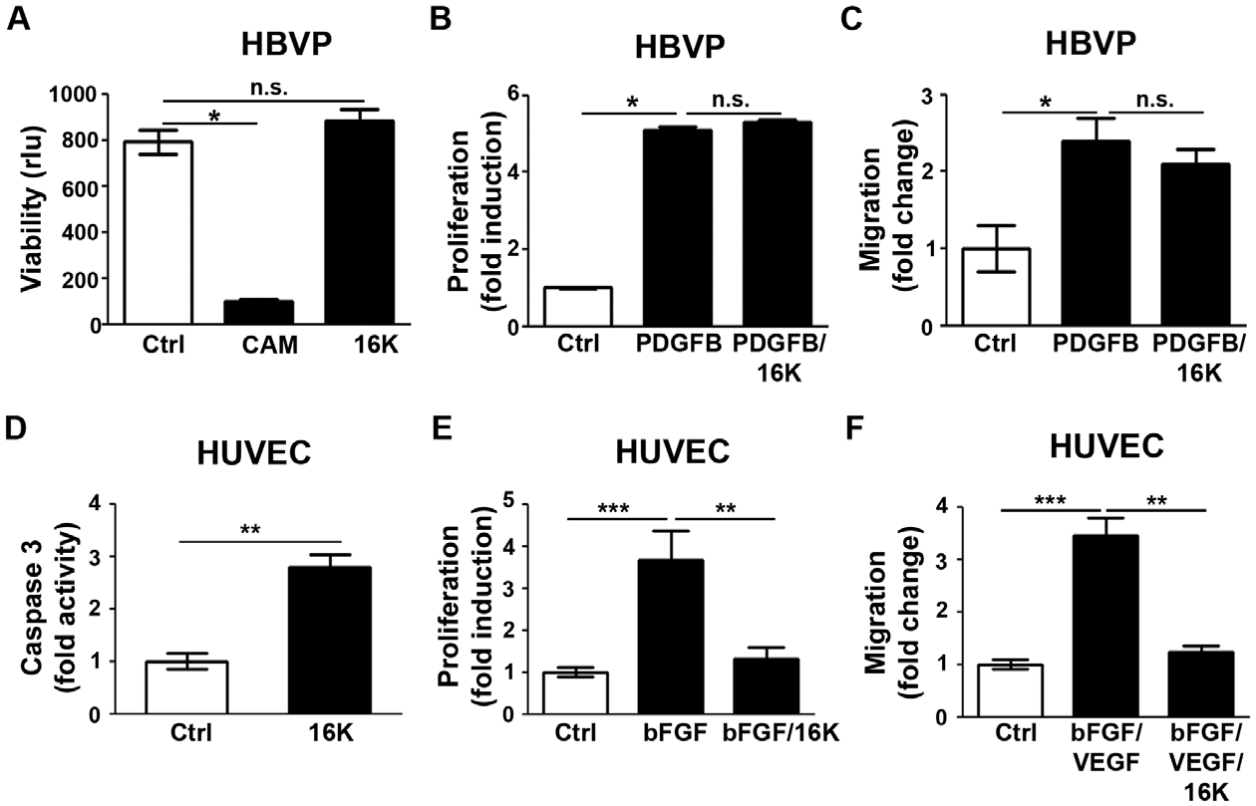
Endothelial cell specific effect of 16K hPRL. The response of human brain vascular pericytes (HBVP) and human umbilical vein endothelial cells (HUVEC) to 16K hPRL was examined. (A) HBVP viability was evaluated by means of the calcein assay. 16K: 100 nM 16K hPRL. Cam: camptothecin-treated cells, a positive control. Rlu: relative luminescence units. Each bar represents a mean ± SEM, n = 3. Two different experiments were performed. (B) HBVP were treated with 20 ng/ml PDGF-BB to stimulate proliferation and 100 nM 16K hPRL (16K). HBVP proliferation was assayed 48 h later by measuring BrdU incorporation. The data presented are means ± SEM, n = 5 and are representative of at least two independent experiments. (C) HBVP migration was assessed in a modified Boyden chamber (Costar, Corning Inc.). HBVP were treated for 17 h with 20 ng/ml PDGF-BB to stimulate migration and 100 nM 16K hPRL (16K). The data presented are means ± SEM, n = 3 and are representative of at least two independent experiments. (D) Apoptosis in HUVEC was assessed by quantification of Caspase-3 activity. HUVEC were treated for 16h with 50 nM 16K hPRL. (E) HUVEC were treated with 10 ng/ml bFGF to stimulate proliferation and 100 nM 16K hPRL (16K). HUVEC proliferation was assayed 24 h later by measuring BrdU incorporation. The data presented are means ± SEM, n = 5 and are representative of at least two independent experiments. (F) HUVEC migration in a scratch-wound assay 8 h after treatment with bFGF and VEGFA and with or without 50 nM 16K hPRL. The data presented are means ± SEM, 5 measurements/condition, n = 3 and are representative of at least two independent experiments. n.s., non significant.*, P<0.05. **, P<0.001. ***, P<0.0001.

Because the described *ex vivo* results might be due to direct effects of 16K hPRL on PC/SMC, we used primary HBVP to confirm that 16K hPRL acts specifically on endothelial cells. 16K hPRL does not affect HBVP viability, in sharp contrast to the topoisomerase I inhibitor camptothecin (Figure 4A). In a BrdU incorporation assay, 16K hPRL treatment does not alter PDGF-BB-induced HBVP proliferation (Figure 4B). In a modified Boyden chamber assay, HBVP migration was increased 2.5-fold when the cells were treated with PDGF-BB; but no direct effect was observed when the HBVP were treated with 16K hPRL (Figure 4C). In contrast, in HUVEC 16K hPRL induces by 3-fold Caspase-3 activity, one of the most common events in the apoptosis signaling pathway (Figure 4D). Furthermore, 16K hPRL inhibits bFGF-induced HUVEC proliferation by 2-fold and migration by 3.5-fold (Figure 4E-F). Together these results strongly argue against any direct effect of 16K hPRL on PC/SMC and confirm its specific effect on endothelial cells.

### 16K hPRL inhibits Notch-related gene expression in endothelial cells and pericytes

**Figure 5:**
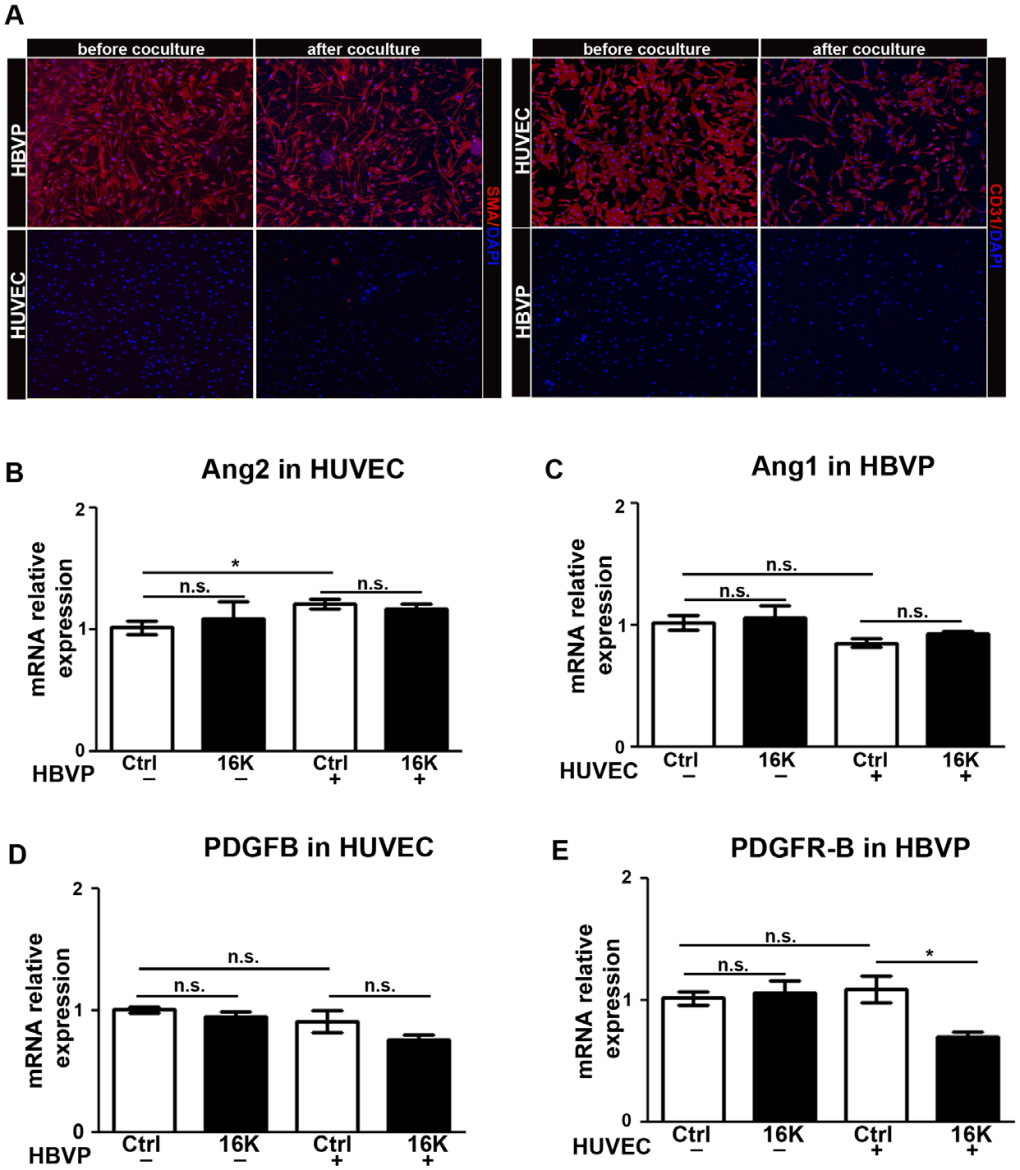
Decreased expression of PDGFR-B in HBVP cocultured with HUVEC after 16K hPRL treatment. A. CD31 (endothelial cells) and α-SMA(pericytes) immunohistochemistry on HUVEC and HBVP cells before and after separation with MACS. (B-E) HUVEC pretreated for 30 min with 100 nM 16K hPRL (16K) were cultured for 16 h in the presence or absence of HBVP. The two cell populations were then separated prior to qRT-PCR analysis and subjected to qRT-PCR analysis to detect *ANG2*, *ANG1*, *PDGFB* and PDGFR-B transcripts. Data are presented as means ± SEM, n = 3 and are representative of at least two different experiments. n.s., non significant. *, P<0.05.

Having observed modified expression of PDGF-B, angiopoietins, Dll4, and EphrinB2 in tumors of 16K hPRL-treated mice, we figured that 16K hPRL might disturb vessel maturation by interfering with the corresponding signaling pathways. To get a clearer picture of the molecular components of endothelial cell-pericyte interaction and of how 16K hPRL affects them, we cultured HUVEC and HBVP alone or together, in the presence or absence of 16K hPRL for 6 h or 16 h. We then separated the endothelial cells from the pericytes with endothelial-cell-specific anti-CD31-conjugated beads. To verify that the isolated cell populations have been successfully purified, we performed immunostaining for specific endothelial cell *vs* pericyte markers before and after cell separation. For both cell types, we did not observe any significant cross contamination (Figure 5A).

We then examined the expression of various markers in the individual cell populations. We first measured *ANG2* and *PDGFB* transcript levels in HUVEC and *ANG1* and *PDGFR-B* transcript levels in HBVP by qRT-PCR. 16K hPRL treatment was not found to have any effect on *ANG2*, *PDGFB* and *ANG1* levels, whether the two cell types were cultured alone or together (Figure 5B–E). Interestingly, 16K hPRL reduced the level of *PDGFR-B* mRNA in HBVP but only when these cells were cultured with HUVEC (Figure 5E).

**Figure 6:**
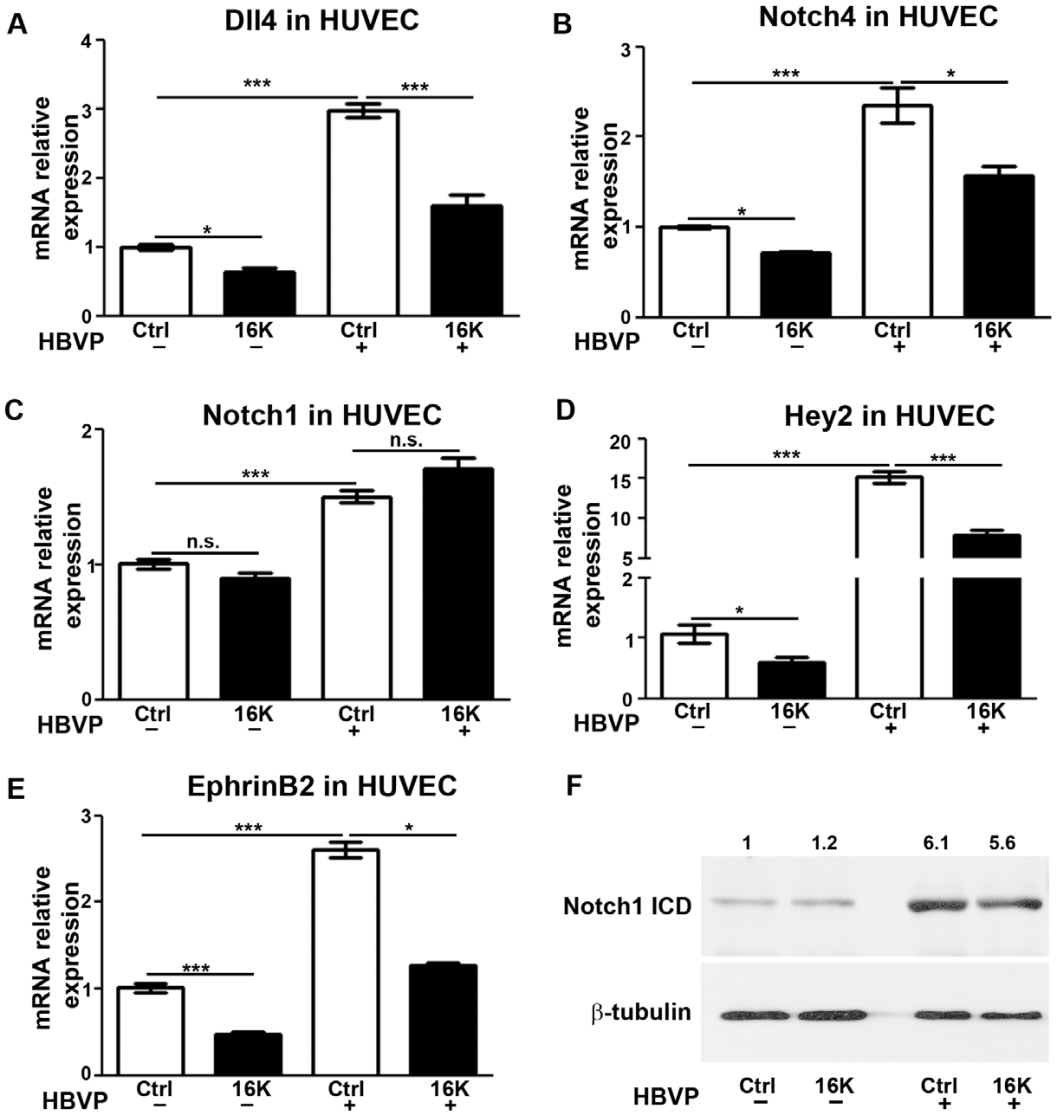
Decreased expression of Notch factors in HUVEC cocultured with HBVP after 16K hPRL treatment. (A–E) HUVEC pretreated for 30 min with 100 nM 16K hPRL (16K) were cultured for 6 h in the presence or absence of HBVP. The two cell populations were then separated prior to qRT-PCR analysis and subjected to qRT-PCR analysis to detect *DLL4*, *NOTCH4*, *NOTCH1*, *HEY2* and *EPHRINB2* transcripts. (F) Western blot analysis to detect Notch1 ICD. Data are presented as means ± SEM, n = 3 and are representative of at least two different experiments. n.s., non significant. *, P<0.05; ***, P<0.0001.

To assess the effect of 16K hPRL on Notch signaling, we focused on transcript-level expression of *DLL4,* the Dll4 receptors *NOTCH1* and *NOTCH4*, and their target genes *HEY2* and *EPHRINB2*. In HUVEC, Notch signaling was activated by coculturing with HBVP, as judged by the increased levels of *DLL4, NOTCH1*, *NOTCH4*, *HEY2* and *EPHRINB2* transcripts (Figure 6A–E). Consistent with this, we observed an elevated level of the intracellular domain of the Notch 1 receptor (Notch1 ICD) which confirms that Notch1 signaling pathway is activated upon coculture of HUVEC and HBVP (Figure 6F). A 6h-treatment with 16K hPRL decreased the levels of *DLL4*, *NOTCH4*, *HEY2* and *EPHRINB2* transcripts in HUVEC cultured alone or with HBVP (Figure 6A, B, D and E) but did not reduce the level of *NOTCH1* mRNA or Notch1 ICD protein (Figure 6C).

**Figure 7:**
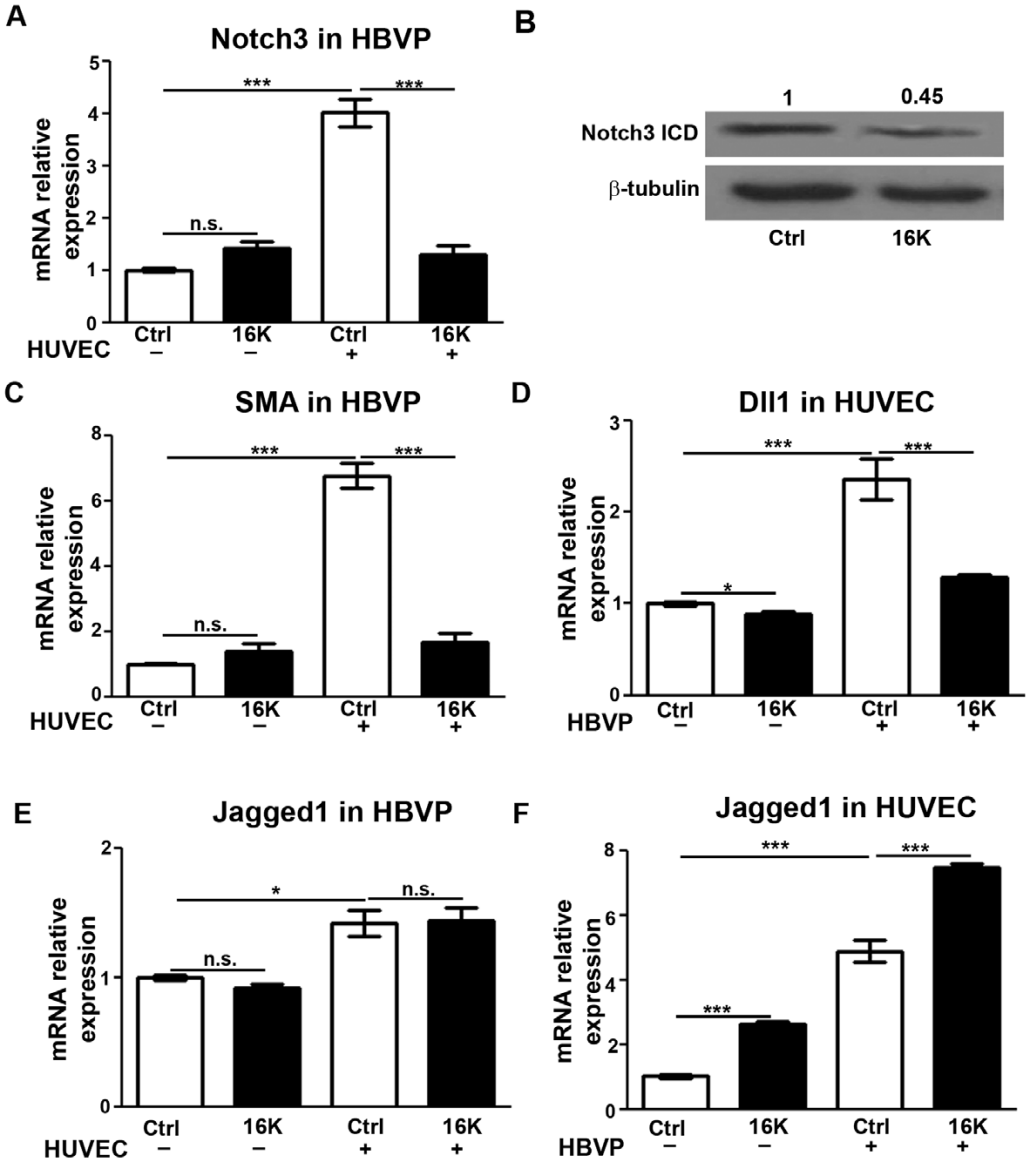
Decreased expression of Notch factors in HUVEC-HBVP cocultures after 16K hPRL treatment. In cultures of HBVP or HUVEC alone, the cells were treated with 100 nM 16K hPRL (16K) for 16 h. HBVP+HUVEC cocultures were seeded and treated as in Fig. 6, but the time spent in coculture was longer (16 h). The two cell populations were then separated prior to qRT-PCR analysis. In HBVP, transcripts of *NOTCH3* and *aSMA* were detected (respectively A and C). Western blot analysis was also performed to detect Notch3 ICD (B). In HUVEC, *DLL1* transcripts were detected (D). In HBVP and HUVEC, *JAGGED1* transcripts were detected (respectively E and F). The presented data are means ± SEM, n = 3 and are representative of at least two independent experiments. n.s., non significant. ***, P<0.0001.

In pericytes, Notch3 also contributes to vessel stabilization through cell-cell contact. Dll1 produced by endothelial cells activates Notch3 on mural cells, thereby promoting mural-cell maturation [24]. In addition, Jagged1 expressed by endothelial cells specifically activates Notch3 in mural cells and this activity is both necessary and sufficient for the expression of smooth muscle genes. Activated Notch3 can promote its own expression as well as the one of its ligand Jagged1 in the same cell [23]. Accordingly, HBVP cocultured with HUVEC produced higher levels of both *NOTCH3* and *aSMA* (smooth muscle marker) transcripts than did HBVP cultured alone (Figure 7A and C). Treatment of the cocultures with 16K hPRL for 16 h significantly decreased the *NOTCH3* and *aSMA* transcript levels in these cells (Figure 7A–C). The decreased level of the Notch3 intracellular domain (Notch3 ICD) detected in pericytes confirms that 16K prolactin reduces the Notch3 signaling in pericytes cocultured with endothelial cells (Figure 7B). In endothelial cells, the level of *DLL1* transcripts was higher in the presence of pericytes (Figure 7D). Interestingly, 16K hPRL treatment caused the *DLL1* transcript level to decrease (Figure 7D). In pericytes and endothelial cells, the level of *JAGGED1* transcripts was higher when they are cocultured together (respectively Figure 7E and F). 16K hPRL treatment did not affect the *JAGGED1* transcript level in pericytes but surprisingly increased its level in endothelial cells.

Together, the results of these expression studies suggest that 16K hPRL affects cell-cell interactions in (co-)cultured endothelial cells and pericytes, notably by interfering with Notch signaling pathways.

## Discussion

We have previously shown in the murine subcutaneous B16-F10 melanoma model that the antiangiogenic factor 16K hPRL reduces tumor growth, although its administration is paradoxically associated with an apparent increase in tumor vessel density [13]. Indeed based on the fact that 16K hPRL exerts *in vitro* its antiangiogenic effects by inhibiting proliferation and inducing apoptosis of endothelial cells, 16K hPRL would be expected to inhibit overall vascular formation. Actually, we previously reported that even if the vessels density is increased, the size of the vessels is strongly reduced [13]. So by inhibiting EC proliferation and inducing their apoptosis, 16K hPRL leads to an increase of vessels number but with a smaller size. This observation could, at least in part, be explained by the effect of 16K hPRL on Notch signaling. Indeed previous studies have shown that tumor treatment with Dll4 inhibitor inhibits tumor growth by promoting non productive angiogenesis. Indeed this blockade led to increased tumor vascularity but with reduced perfusion [32]. Similarly in this study we observed that 16K hPRL treatment leads to an increased vessel number but the vessel perfusion is reduced.

Furthermore, the tumor blood vessels observed after treatment (contrarily to other vessels) displayed reduced coverage by pericytes. This is a sign that the vessels failed to mature properly. Our data thus suggest that 16K hPRL treatment prevents the development of a normally functioning tumor vasculature by impairing vessel maturation. Our observations are in line with several studies on tumor vessel maturation in mouse models, where reduced pericyte recruitment is associated with increased vessel density but decreased tumor vessel perfusion [33], [34], [35]. In such situations, reduced pericyte recruitment may contribute to excessive vessel sprouting. The result is a highly chaotic vascular network lacking the hierarchy essential to efficient directional blood flow.

Further evidence that 16K hPRL affects vessel maturation relies on the ability of 16K hPRL to inhibit outgrowth of a pericyte/smooth muscle cell network *ex vivo* and pericyte recruitment to endothelial cells *in vitro*. These effects on pericyte behavior appear to be indirect, since 16K hPRL shows no toxicity towards these cells and does not inhibit their proliferation or ability to migrate.

Various pathways and factors involved in pericyte recruitment during embryonic development have also been linked to tumor angiogenesis. One of these factors is PDGF-B. Tumors engineered to overexpress PDGF-B show an increased coverage of blood vessels by pericytes [36], [37]. Inhibition of PDGFR-B signaling can loosen, detach, or even eliminate pericytes from the tumor vessels [38]. The role of the development-related factors Ang1 and Ang2 in tumor-associated angiogenesis remains controversial. Blood vessel stabilization by Ang1 might either promote tumor angiogenesis or reduce tumor growth depending on the tumor model [20]. Ang2 is an antagonist of Ang1. It is only weakly expressed in endothelial cells under physiological conditions, but its expression is dramatically increased during vascular remodeling, e.g., during tumor growth. Increased levels of Ang2 in the tumor endothelium have been shown to result in aberrant vessels with poor smooth muscle cell coverage [20]. We observed reduced *PDGF-B* and *ANG1* expression and increased *ANG2* expression in the tumors of 16K hPRL-treated animals. This, according to the literature, strengthens our hypothesis that 16K hPRL restricts tumor vessel maturation. Yet we observed no transcript-level deregulation of these factors in our *in vitro* cocultures. This may be due to the fact that culture conditions do not perfectly mimic the *in vivo* tumor microenvironment, but we cannot exclude a contribution of the tumor compartment to the mRNA levels measured in whole tumor tissue. However, we observed a reduced *PDGFR-B* expression in pericytes cocultured with endothelial cells after 16K hPRL treatment.

In addition to the above-mentioned paracrine factors, molecules involved in direct cell-cell contact are also essential to vessel maturation. Recently, the Notch signaling pathway has emerged as indispensable to vessel maturation through its role in pericyte recruitment and cell-cell-contact-mediated differentiation [17]. We therefore hypothesized that disruption of the Notch signaling pathway might contribute to explaining our results. In support of this hypothesis, we show that in endothelial cells cultured alone or with pericytes, 16K hPRL decreases expression of the ligand Dll4, its receptor Notch4, and the target genes Hey2. Furthermore, Dll4 expression is diminished in tumors after 16K hPRL treatment. These results are in line with those of Scehnet *et al.*, who demonstrated decreased growth of S180 tumors and defective maturation of tumor vessels in response to inhibition of Dll4 signaling. In their work, the tumor vessels displayed a small caliber, a reduced vessel lumen, markedly reduced pericyte recruitment, and deficient vascular perfusion [35]. This is precisely the phenotype that we have observed here. In addition, Real and collaborators have recently showed that transplantation of bone marrow-derived vascular precursor cells with decreased Dll4 into tumor-bearing mice resulted in the formation of unstable vessels, as evidenced by the reduced pericyte coverage and reduced Fibronectin expression [39]. This led to a decreased tumor growth. All this supports the above hypothesis.

**Figure 8:**
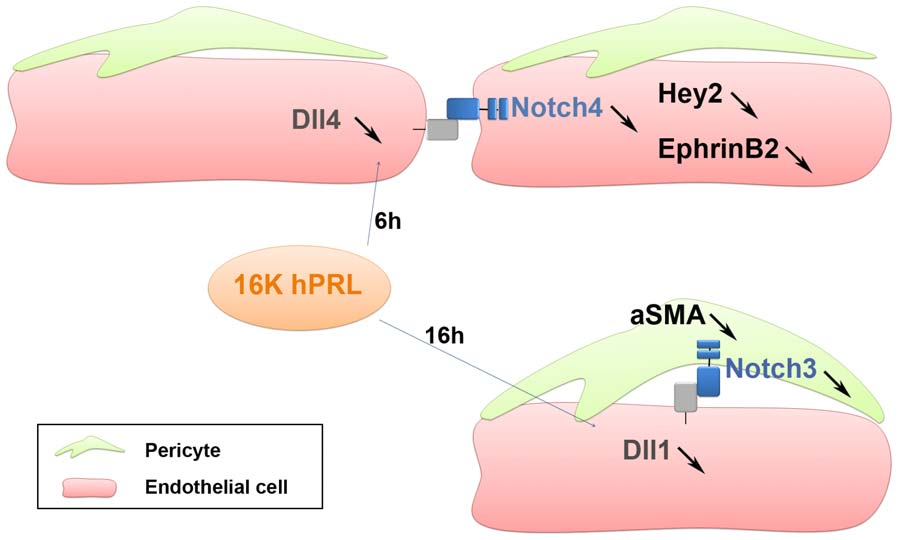
Hypothetical model of 16K hPRL effects on Notch signaling in endothelial/pericyte interactions. When endothelial cells are cultured with pericytes, Dll4/Notch4 pathway is activated. 16K hPRL treatment (6 h) alters Dll4/Notch4 signaling by decreasing *DLL4*, *NOTCH4*, *HEY2* and *EPHRINB2* transcripts in endothelial cells. After 16 h of endothelial cell-pericyte cocultures, Notch3 and Dll1 are increased respectively in pericytes and endothelial cells. 16K hPRL inhibits the Dll1/Notch3 signaling by reducing *DLL1* transcripts in endothelial cells and *NOTCH3* and *aSMA* transcripts in pericytes.

Recently, an essential role in post-natal assembly of pericytes with endothelial cells has been attributed to EphrinB2, which also plays a crucial role in non-productive angiogenesis caused by a Dll4 blockade [28], [40]. Interestingly, we show that EphrinB2 expression is decreased by 16K hPRL both in 16K-Ad-treated tumors and in endothelial cells cultured alone or with pericytes. These data suggest that an angiogenesis inhibitor like 16K hPRL can impair the assembly of pericytes with endothelial cells by perturbing Dll4/Notch4 crosstalk and EphrinB2 expression (see model on figure 8).

Notch signaling is not restricted to endothelial cells. Pericytes and vascular smooth muscle cells express mainly the receptor Notch3, a key player in vessel maturation [22]. Here, we show that coculturing with endothelial cells increases *NOTCH3* and *αSMA* expression in pericytes. Conversely, we have measured the level of transcripts encoding Dll1, a ligand of Notch3, in cultured endothelial cells, showing that it rises in response to coculturing with pericytes. These results are in agreement with a recent study showing that endothelial cells induce mural cell differentiation through activation and induction of Notch3 [23]. Interestingly, 16K hPRL treatment caused reduced *DLL1* expression in endothelial cells and reduced *NOTCH3*, NOTCH3 ICD and *αSMA* expression in pericytes cocutured with endothelial cells. Since 16K PRL does not act directly on pericytes, our results suggest that the reduced expression of Dll1 by 16K hPRL in endothelial cell would lead to a decrease in the expression of the Notch3 receptor in pericytes. Consequently the Notch3 signaling is reduced in pericytes resulting in a decrease level of smooth muscle gene transcription in these cells (see model on Figure 8). These results suggest that 16K hPRL can hinder the differentiation of pericytes which may be responsible of the reduced pericytes coverage of blood vessels observed in tumors treated by 16K hPRL.

The regulation by 16K hPRL of another Notch ligand Jagged1 is interesting. As previously shown by others, we observed that *JAGGED1* levels are elevated when pericytes are cocultured with endothelial cells [21]. Surprisingly, *JAGGED1* levels are increased in endothelial cell following 16K hPRL treatment. Although this could appear paradoxical, this type of regulation has previously been observed for other factors. For example, TNF-alpha decreased *Dll4* expression in endothelial cells while increased *Jagged1* expression [41]. This observation is consistent with the opposite effects of Dll4 and Jagged1 in angiogenesis previously reported [41].

The mechanisms by which 16K hPRL acts on the above-mentioned vessel-maturation-related factors and pathways are under investigation. At present, we know that 16K hPRL inhibits VEGF-induced Ras activation, thereby blocking MAPK activation and abolishes bFGF-stimulated growth of cultured endothelial cells. Interestingly, VEGF enhances *PDGFB* expression [42], and VEGF and bFGF are positive regulators of Dll4 and EphrinB2 expression in endothelial cells [43]. We might thus speculate that inhibition of VEGF and bFGF signaling by 16K hPRL leads to inefficient activation of the PDGF-B/PDGFR-B, Ang/Tie2, and Dll4/Notch pathways. In addition, as Dll4 signaling from endothelial cells to Notch-expressing mural cells upregulates PDGFR-B expression [44], we might speculate that 16K hPRL by regulating Dll4 expression affects PDGFR-B signaling and thereby inhibits vessel maturation. We might also speculate that 16K hPRL affects the Delta/Notch pathway by regulating NF-κB, since 16K hPRL has been shown to activate NF-κB in endothelial cells [6] and since complex cross-talk between NF-κB and Notch has been described at multiple levels in vertebrate systems [45].

To date, four angiogenesis inhibitors have been approved for cancer treatment by the U.S. Food and Drug Administration [46]: bevacizumab, sorafenib, sunitinib, and thalidomide. These antiangiogenic drugs that disrupt VEGF signaling, induce a transient functional normalization of the tumor vasculature (immature vessels are pruned, blood vessel tortuosity decreases, and a closer association between pericytes and endothelial cells is induced) that can improve the activity of coadministered chemotherapies or the effect of radiation therapy [47], [48]. Molecularly, VEGF inhibition causes vessel normalization in part by the upregulation of Ang1 and by the activation of the PDGFR-B signaling that enhance pericyte coverage of the vessels [44]. Our data suggest that 16K hPRL does the opposite. This is quite interesting in the light of the fact that protection of the tumor vasculature by increased pericyte coverage is one of emerging mechanisms of evasive resistance to antiangiogenic therapies [49]. Recently, Helfrich *et al.* have shown that tumor vessels resistant to anti-VEGF therapy are characterized by an enhanced vessel diameter and by normalization of the vascular bed by coverage with mature pericytes [50]. Clinical trials are underway to determine whether it is preferable to inhibit a single pathway (and cause transient normalization) or to combine VEGF inhibitors with agents that disturb vascular “normalization” by disrupting pericyte support [49]. Activation of the Notch ligand/receptor system plays an important role in the development of resistance to antiangiogenic therapy due to increased vessel maturation and normalization [46]. Tumors with intrinsic resistance to anti-VEGF agents appear to be sensitive to inhibition of Dll4 [46], [51]. The association between Dll4 and tumor resistance to anti-VEGF inhibition suggests that targeting Notch factors in addition to VEGF may prove to be of greater therapeutic effect [51].

Here, we show that the antiangiogenic agent 16K hPRL impairs tumor vessel maturation by reducing activation of the Notch ligand/receptor pathway. This is the first time, to our knowledge, that an endogenous antiangiogenic compound is described to inhibit Notch signaling in endothelial cells and pericytes. In the light of all these data, 16K hPRL appears as a very promising agent for future antiangiogenic cancer therapy with a lesser likelihood of treatment resistance linked to vessel maturation and stabilization.

## Materials and Methods

### Ethics Statement

The human endothelial cells used in this study were generated from umbilical cords obtained of women donors after delivery. A written informed consent was obtained from each individual. The human protocols were approved in accordance to legal provision by the Ethics Committee of the “Centre Hospitalier Régional Citadelle”. All animal experiment protocols were approved by the Institutional Animal Ethics Committee of the University of Liège under application number 941.

### Production of recombinant proteins

Recombinant 16K hPRL was produced in *E. coli* as previously described [6]. The purity of the recombinant protein exceeded 95% (as estimated by Coomassie blue staining) and the endotoxin level was 0.0005 ng/ng recombinant protein, as quantified with the rapid endo test of the European Endotoxin Testing Service (BioWhittaker, Verviers, Belgium).

### Adenovirus vectors

16K-Ad is a defective recombinant E1-E3-deleted adenovirus vector encoding a secreted peptide consisting of the first 139 amino acids of PRL. This adenovirus vector was constructed as described [10], using the Adeno-X expression system (BD Biosciences, Erembodegem, Belgium). Purification and titration of the recombinant adenoviruses were performed respectively with the BD Adeno-X Virus Purification kit (BD Biosciences, Erembodegem, Belgium) and the Adeno-X Rapid Titer kit (BD Biosciences, Erembodegem, Belgium), according to the manufacturer's instructions. Null-Ad is a control adenovirus carrying an empty expression cassette.

### Quantitative real time-polymerase chain reaction (qRT-PCR)

Total RNA was isolated with the RNeasy mini kit (Qiagen, Venlo, Netherlands) or the Nucleospin RNA/Protein kit (Macherey-Nagel GmbH, Dueren, Germany) and reverse transcribed with the iScript cDNA Synthesis kit (Bio-Rad, Hercules, CA, USA) to generate cDNA. Quantitative PCR was performed using a one-step system (Applied Biosytems, Carlsbad, CA, USA) with Faststart Universal SYBR Green Master (Roche, Clinical Laboratories, Indianapolis, IN, USA). The fold difference for various transcripts was calculated by the ΔΔCT method using cyclophilin A (PPIA) as the internal control. The following primers were used for qRT-PCR: human ANG1 forward (5′-CTCGCTGCCATTCTGACTCAC-3′), reverse (5′-GACAGTTGCCATCGTGTTCTG-3′); murine ANG1 forward (5′-CTCAGTGGCTGCAAAAACTTGA-3′), reverse (5′-TGGCCGTGTGGTTTTGAAC-3′); human ANG2 forward (5′-TAAGCAGCATCAGCCAACCAG-3′), reverse (5′-AAACCACCAGCCTCCTGTTAGC3′-); murine ANG2 forward (5′-ACCTTCAGAGACTGTGCGGAAA-3′), reverse (5′-CGTCCATGTCACAGTAGGCCTT-3′); murine PDGFB forward (5′-CCATCCGCTCCTTTGATGAT-3′), reverse (5′-TCAGCCCCATCTTCATCTACG-3′); human PDGFB forward (5′-AACAACCGCAACGTGCAGT-3′), reverse (5′-GTCACCGTGGCCTTCTTAAAGA-3′); human PDGFR-B forward (5′-AGACACGGGAGAATACTTTTGC-3′); reverse (5′-AGTTCCTCGGCATCATTAGGG-3′); human Jagged1 forward (5′-TGGGCTTTGAGTGTGAGTGT-3′); reverse (5′-CCCCGTGGGAACAGTTATTA-3′); human DLL4 forward (5′- ACCACTTCGGCCACTATGTGTG -3′), reverse (5′-AGCCCGAAAGACAGATAGGCTG-3′); human DLL1 forward (5′-ATATGCCCCAACGAATGCTG-3′), reverse (5′-GCTCGGTCTGAACTCGGTTTC-3′); human Notch1 forward (5′-CGGGTCCACCAGTTTGAATG-3′), reverse (5′-GTTGTATTGGTTCGGCACCAT-3′); human Notch4 forward (5′-CGATAATGCGAGGAAGATACGG-3′), reverse (5′- GATCGGAATGTTGGAGGCA-3′); human Notch3 forward (5′-CAATGCTGTGGATGAGCTTG-3′), reverse (5′-AAGTGGCTTCCACGTTGTTC-3′); human HEY2 forward (5′-TGAGTTGAGAAGACTTGTGCCA-3′), reverse (5′-AAGTAGCCTTTACCCCCTGTTG-3′); human EphrinB2 forward (5′-CGTGCTGGAGAATTGACTGA-3′), reverse (5′-CTGTCTTCCCTTGGCTTCTG-3′); human aSMA forward (5′-CAAGTGATCACCATCGGAAATG-3′), reverse (5′-GACTCCATCCCGATGAAGGA-3′); murine PPIA forward (5′-ACCGTGTTCTTCGACATCACG-3′), reverse (5′-CTGGCACATGAATCCTGGAATA-3′); human PPIA forward (5′-CCAACACAAATGGTTCCCAGT-3′), reverse (5′-CCATGGCCTCCACAATATTCA-3′).

### Mice

Adult female C57BL/6J mice (6–8 weeks of age) purchased from the Central Animal Facility of CHU Liège (Liège, Belgium) were used to assess tumor growth.

### *In vivo* tumorigenicity

C57BL/6 mice were i.v. inoculated with either 10^9^ pfu Null-Ad or 10^9^ pfu 16K-Ad adenovirus vector. Two days later, mice were *s.c.* injected with 10^5^ B16-F10 mouse melanoma cells. Adenovirus vector injection was repeated once, one week after the first inoculation. Fifteen days after tumor inoculation, the mice were sacrificed and their tumors harvested. Tumor volumes were measured every one or two days and calculated as follows: length x width^2^×0.5 [52].

### Evaluation of vessel perfusion

To mark vessel perfusion, tumor-bearing mice were i.v. injected with FITC-*Lycopersicon esculentum* lectin in 0.9% NaCl (100 µg, Vector Laboratories, Burlingame, CA, USA) 3 minutes before sacrifice and perfused with 1% paraformaldehyde (PFA). Tissues were removed, postfixed in 1% PFA for 1 hour at 4°C, incubated overnight in 30% sucrose, and snap frozen. A perfusion index was quantified as the percentage of lectin-positive vessels per CD31-positive vessels in the B16F10 tumors.

### Tracheal analysis

Before sacrifice, mice were injected with FITC-*Lycopersicon esculentum* lectin and perfused with 1% PFA. Tracheas were removed, immersed in fixative for one hour, and stained as whole mounts. Pericytes were stained with rabbit anti-mouse desmin (clone Y66, 1:1000, Millipore, Bedford, MA, USA) followed by a secondary Cy3-conjugated antibody (Jackson ImmunoResearch, West Grove, PA, USA). Digital images were acquired with a fluorescence microscope (Eclipse 90i, Nikon Belux, Bruxelles, Belgium). The pericyte density in the cartilaginous region (capillary region) was measured in at least 5 regions per animal with ImageJ software (http://rsbweb.nih.gov/ij/).

### Immunohistochemistry

To assess vessel maturation, frozen tumor sections (6–80-µm thick) were fixed in acetone (VWR, Leuven, Belgium) for 30 min at 4°C. Next, the sections were incubated with a rat monoclonal anti-CD31 (1/250, BD Biosciences, Erembodegem, Belgium) for 2.5 h, followed by a biotinylated donkey anti-rat secondary antibody (1/100, Jackson immunoresearch, Suffolk, UK) and finally by streptavidin/Cy5 complex (1/150, Jackson Immunoresearch, Suffolk, UK) for 1.5 h at room temperature. These CD31-positive sections were incubated for 2.5 h at room temperature with Cy3-conjugated mouse monoclonal anti-αSMA (1/1500, Sigma-Aldrich, Bornem, Belgium) or with rabbit polyclonal anti-desmin (1/1000, Abcam, Cambridge, UK) or rabbit polyclonal anti-NG2 (1/200, Millipore, Bedford, MA, USA) followed by incubation with goat anti-rabbit IgG-Cy3 (1/500, Jackson Immunoresearch, Suffolk, UK) for 1.5 h at room temperature. To verify that the isolated cell populations have been successfully purified, we performed immunostaining for specific endothelial cell vs pericyte markers before and after cell separation. After separation, cells were seeded on coverslips. After 24 h, cells were fixed for 30 min with 1% PFA in PBS and incubated with the blocking solution (20% FBS, 1% BSA) for 15 min. Cells were then incubated with a mouse anti-human CD31 (1/50, BD Biosciences, Erembodegem, Belgium) or with a mouse anti-human α-Smooth Muscle Actin (1/50, Sigma-Aldrich, Bornem, Belgium) for 1 h, followed by incubation with a biotinylated polyclonal goat anti-mouse antibody (1/50, Jackson immunoresearch, Suffolk, UK) and with a streptavidin-PE antibody (1/50, Jackson immunoresearch, Suffolk, UK). The samples were mounted with Prolong (Invitrogen Corp./Life Technologies, Paisley, Scotland). All immunohistochemical stainings were examined with a standard fluorescence microscope (90i Eclipse, Nikon Belux, Bruxelles, Belgium).

### Image processing and measurement of tumor pericyte coverage

Gray level images of vessels and covered regions were binarized automatically using the entropy of the histogram of gray level intensities [53]. On the binarized images obtained, the area occupied by vessels and the covered area were estimated. Coverage was calculated by dividing the latter area by the former. At least 15 images per tumor were analyzed. For statistical analysis, the unpaired Wilcoxon test was used to compare the Null-Ad- and 16K-AD-treated groups. Results were considered significant at *P*<0.05.

### *Ex vivo* aortic ring assay

Mouse aortic rings were cultured in three-dimensional type I collagen gels as previously described [54], [55]. Effects of different compounds were tested by adding them to the culture medium on day 0. For quantification, computerized image analysis was performed on a Sun SPARC30 workstation with the Visilog 5.0 (Noesis) software according to Blacher *et al.* [56]. A grid of concentric rings is used to determine the number of intersections with sprouting cells. Results are expressed as number of intersections plotted as a function of the distance to the ring.

### Aortic ring whole-mount immunostaining

On day 9 of culture, aortic fragments embedded in collagen gels were washed in PBS and fixed in 4% PFA. Nonspecific antibody binding was blocked with 1.5% milk after washing in PBS. Samples were incubated overnight at room temperature with a mix of lectin and primary antibody: Griffonia Simplifolia isolectin-B4/Alexa Fluor 488 at 5 µg/ml (I21411, Invitrogen Corp./Life Technologies, Paisley, Scotland) and rabbit anti-NG2 chondroitin sulfate proteoglycan antibody (AB5320, Millipore, Bedford, MA, USA) at 10 µg/ml. After washing with PBS, the tissues were incubated at room temperature with a secondary biotinylated goat anti-rabbit antibody (E432, Dako, Glostrub, Denmark) at 1.9 µg/ml. This was followed by a 1-h incubation with 1 µg/ml streptavidin-Cy3 (S6402, Sigma-Aldrich, Bornem, Belgium). Samples were mounted in Vectashield-Dapi mounting medium (H-1200, Vector Laboratories, Burlingame, CA, USA).

### Cell cultures

HUVEC (Human Umbilical Vein Endothelial Cells) were isolated as previously described [57]. They were cultured in EBM2 supplemented with 5% FCS and the EGM-2 bullet kit at 37°C in a 5% CO_2_ humid atmosphere (Lonza, Walkersville, MD, USA). HBVP (Human Brain Vascular Pericyte) cells were purchased from Sciencell research laboratories (Sciencell research laboratories, Carlsbad, CA, USA) and cultured in PM (pericyte medium) supplemented with 2% FCS, 10% pericyte growth supplement and 100 units/ml penicillin, 100 µg/ml streptomycin at 37°C in a 5% CO_2_ humid atmosphere (Sciencell research laboratories, Carlsbad, CA, USA). B16-F10 mouse melanoma cells were obtained from the American Type Culture Collection (ATCC CRL-6475, Rockville, Md, USA) and cultured in high-glucose DMEM supplemented with 10% FCS and 4 mM glutamine, 100 units/ml penicillin, 100 µg/ml streptomycin at 37°C in a humid 5% CO_2_ atmosphere (Invitrogen Corp./Life Technologies, Paisley, Scotland).

### Under-agarose migration assay

Endothelial cells and pericytes were plated in two 5-mm wells created 1 mm apart in 1% agarose/1% FCS in EBM2. Twenty thousand HUVEC were plated in one well in 25 µl EBM2 with 1% FCS. After 3 hours the HUVEC were treated with 100 nM 16K hPRL, and 30 min later HBVP (2×10^4^ cells in the same medium) were added to the other well. The HUVEC and HBVP were incubated for 72 h. The cells were then fixed with 2% PFA in PBS for 30 min and stained with 1% Coomassie blue overnight. The distance of HBVP migration towards the HUVEC was measured with ImageJ software. The experiments were performed in duplicate and the data for statistical analyses were taken from three independent sets of experiments.

### Viability assay

HBVP cells were plated at a density of 2×10^4^ cells per well (in 24-well plates) in 0.5 ml pericyte medium. The cells were treated with 100 nM 16K hPRL or 10 µM campthotecin (Sigma-Aldrich, Bornem, Belgium) for 72 h. After washing with PBS and incubation with 1 µM calcein-AM (Calbiochem, Nottingham, UK) for 30 min at RT, cell fluorescence was measured at 535 nm with a microplate reader (Wallac Victor^2^, Perkin Elmer, Norwalk, Finland).

### Caspase-3 activity assay

HUVEC were plated in 48-well culture plates at a density of 50×10^3^ cells per well in 500 µl of culture medium. After 48 h incubation, the cells were treated with 50 nM hPRL 16K. Caspase-3 activity was measured 16 h post-treatment with the CaspACE Assay System Fluorimetric (Promega Corp., Madison, WI) according to the manufacturer's instructions.

### Proliferation assay

HBVP were plated in 96-well culture plates at a density of 2×10^3^ per well in pericyte medium. After overnight incubation, the culture medium was replaced with fresh medium containing 0.5% FCS and incubated for 24 h. The medium was then replaced with serum-free pericyte medium and the cells were stimulated with 20 ng/ml PDGF-BB (R&D Systems Europe Ltd, Oxon, UK) in the presence or absence of 100 nM 16K hPRL. Proliferation was analyzed 48 h later by measuring BrdU incorporation with the Cell Proliferation ELISA, BrdU (Colorimetric) (Roche Clinical Laboratories, Indianapolis, USA). HUVEC were plated in 96-well culture plates at a density of 7×10^3^ per well in their culture medium. After 48 h incubation, the cells were stimulated with 10 ng/ml bFGF (Promega Corp.) in the presence or absence of 50 nM 16K hPRL. Proliferation was analyzed 24 h later by measuring BrdU incorporation with the Cell Proliferation ELISA, BrdU (Colorimetric) (Roche Clinical Laboratories, Indianapolis, USA).

### Modified Boyden chamber migration assay

HBVP cell migration was assessed by means of a modified Boyden chamber assay. Polycarbonate filters (8 µm pore) from Transwell® (Costar Corp, Cambridge, MA.) were treated overnight at room temperature with 1% gelatin. The cells were trypsinized, resuspended in serum-free medium containing 0.1% BSA, and placed in the upper compartment of the chamber (80×10^3^ cells/filter). The lower compartment of the chamber was filled with medium containing 1% FCS and 1% BSA, supplemented with 20 ng/ml PDGF-BB in the presence or absence of 100 nM 16K hPRL. After incubation at 37°C for 17 h, the filters were fixed in methanol. Cells on the filters were stained with 4% Giemsa solution (Fluka Chemika, Buchs, Germany). Cells that had not migrated, at the upper surface of the filters, were wiped away with a cotton swab. Migration was quantified by counting the number of cells on the lower surface of the filters.

### Scratch wound migration assay

HUVEC (80×10^3^ cells) were seeded into a 48-well plate in culture medium and incubated for 48 h to reach confluence. With a tip, a wound was made in the monolayer (at time 0). The cells were then washed with PBS and stimulated with different media. To favor migration, cells were incubated for 8 h with medium containing both 10 ng/ml recombinant bFGF (Promega Corp.) and 50 ng/ml recombinant VEGFA (RELIATech GmbH) in the presence or absence of 50 nM 16K hPRL. The distance between the two sides of the wound was measured with a graduated ocular lens coupled with an Olympus CKX41 microscope (Olympus). The distance between the two sides of the wound after 8 h of migration was divided by the distance at time 0 and represented on a graph.

### Cocultures with direct contact

For 2-dimensional cocultures, 6×10^5^ HUVEC were plated in EBM2 with 1% FCS and 30 ng/ml VEGF in a T25 flask. After adhesion, 6×10^5^ HBVP were added. 16K hPRL was added 30 min prior to the addition of HBVP in the indicated experiments. To separate the endothelial cells and pericytes, anti-CD31-conjugated MicroBeads (Miltenyl Biotec GmbH, Bergisch Gladbach, Germany) were used according to the manufacturer's instructions. The separate cell populations were then processed for western blot analysis or quantitative RT-PCR.

### Western blot analysis

Equivalent amounts of proteins from tumor tissues or cells were resolved by SDS-PAGE and transferred to polyvinylidene fluoride membranes (Millipore, Bedford, MA, USA). The membranes were saturated for 1 h in TBS-8% dry milk, then incubated for 1 h with primary antibodies and for 1 h with secondary antibodies. Detection was carried out by chemiluminescence with the ECL Plus kit (Amersham Biosciences, Arlington Heights, IL, USA). Primary antibodies: DLL4 and NOTCH3 (Santa Cruz Biotechnology, Santa Cruz, CA USA), NOTCH1 ICD and EPHRINB2 (Millipore, Bedford, MA, USA), TUBULIN (Abcam, Cambridge, UK).

### Statistical analysis

All data are expressed as means ± SEM. Analyses for statistical significance (the Mann-Whitney test) were carried out with Prism 5.0 software (GraphPad Software, San Diego, CA, USA). Statistical significance was set at *P*<0.05.
